# Our Journal and Its Objects

**Published:** 1877-01-20

**Authors:** 


### Editorial and Miscellaneous.

### OUR JOURNAL AND ITS OBJECTS.

As the SOUTHFRN MEDICAL RECORD, in its present new dress, will be sent to many who have not seen it, or been made acquainted with its objects, it seems not inappropriate that we say something in this issue about the plan and designs of our journal.

In inaugurating the enterprise, several years ago, we were well aware of the fact that failure had resulted from every similar undertaking in the Southern States. So uniformly, indeed, had this been the case that the charge had been often made, and received as a commonly accepted fact, that the physicians of the South could not be depended upon to support a medical journal.

In reflecting upon the cause of these repeated failures, the fact presented itself to our mind that the practitioners of the great South and West *had never been furnished with a journal adapted to their wants.* The large majority of them residing in remote and sparsely settled regions of country, and being much of the time in the saddle, need a journal practical rather than theoretical; and as they seldom take but one journal, they desire one that will give the pith and cream of all that is new, practical and useful in the medical world, in as few words as possible. This being true, it is not surprising that the Southern journals have not been acceptable, as in nearly every instance they were issued in the interest of the medical colleges, and have devoted much of their space to introductory lectures, and long, prosy dissertations, designed to advertise the schools and the writers rather than to benefit the busy practitioner.

From these considerations we determined to issue a journal, the leading idea of which should be brevity in the publication of the truly useful and practical in the medical progress of the age. We confess that we have not as yet fully attained to our ideal, but we have approximated it very nearly. The result is that we have a large and increasing list of subscribers, and a constant demand for the back volumes of THE RECORD, which we are assured constitutes the best and most complete abstract of the medical news and information of the last seven years, to be found anywhere in Europe or America.

Our present new issue is in furtherance of the same leading object to which we have alluded, and, we trust, will accomplish it. The reduction in price, demanded by the pressure of the times, could only be attained by the reduction in the size of our journal. And yet we think the present number will show that the value of the RECORD will not be lessened, but rather enhanced by the change, and that the editors, by increased attention and diligence, can compress into its pages an equal, if not a greater amount of useful and practical matter than was heretofore done.

